# 

**Published:** 1892-02-13

**Authors:** 


**Notices of Births, Deaths, and Marriages**, are inserted in this column at a charge of 2s. 6d. for an announcement not exceeding four lines.

### NOTICE TO CORRESPONDENTS.

All MS., Letters, Books for Review, and other matters intended for the Editor should be addressed THE EDITOR, THE LODGE, PORCHESTER SQUARE, LONDON, W.

The Editor cannot undertake to return rejected M.S., even when accompanied by stamped directed envelope.

### Notice to Advertisers and Subscribers.

All Advertisements, Orders for Copies of THE HOSPITAL, and Business Communications should be addressed to The Publisher, *not to the Editor*, THE HOSPITAL, 140, Strand, W.C.

The Cost of Subscription, post free, is as follows:—Per week, 1½d.; per quarter, 1s. 8d.; per half-year, 3s. 3d.; per annum, 6s. 6d. Foreign:—Per annum, 8s. 8d.; payable, in all cases, in advance.

### "Burdett's Hospital Annual," 1891—1892.

Third year of publication. 600 pp. Boards, gilt lettering. A most complete guide to British and Colonial Hospitals, Dispensaries, Nursing and Convalescent Institutions, and Asylums. Price 3s. 6d. Published by THE HOSPITAL (Limited), 140, Strand, W.C.

**NOTICE.—The Index for Vol. X. (ending September, 1891), is on sale at the Office of this Journal, price 2½d., post free.**

### GENERAL CHARITIES.

[This Column is devoted to an account of work of charitable institutions other than Medical Charities. The Editor will be glad to receive reports or news of work at 140, Strand. *Philanthropy* should be plainly written on the envelope.]

Lady Arthur Hill has recently joined the London Committee of the Irish Distressed Ladies' Fund, which has the active support in Ireland of the Countess of Zetland and Lady Wolseley. The oldest pensioner on the fund died last month, at the advanced age of ninety-seven, and during the past year eight of the hundred and four ladies in receipt of pensions from the fund died.

For the sake of the Gordon Boys' Home at Chobham and of the British Home for Incurables, Clapham Rise, we hope that the sermon preached by Canon Fleming, at Sandringham, on the Sunday after the Duke of Clarence and Avondale's funeral, which has recently been published, will have a large sale. By the desire of the Princess of Wales, who has added to the sermon a touching anecdote connected with the Prince's death, all the profits arising from the sale will be given to these two institutions.

The Food and Shelter Society appeals for funds. The objects of the Society are mainly threefold : To give immediate assistance in the shape of food and shelter to those who are in actual need of such assistance; to give pecuniary assistance where it is perfectly clear that such assistance is likely to benefit permanently the recipients; and to aid young girls, who have been persuaded to leave their homes, to return to their parents. The Society wisely refrains from immediate gifts of money, but distributes to those who are found to be in want tickets which entitle the holders to food or to shelter. Miss Sheern, the Secretary, will will glad to receive the names of voluntary workers as well as donations at 181, Victoria Street, S.W.

Mr. Alderman Dimsdale has been elected one of trustees of the **CITY OF LONDON TRUSS SOCIETY**, in the room of the late Sir Robert Fowler. During the past year 9,584 patients have been relieved by this society, and 9,632 instruments were supplied. The amount of renewed annual subscriptions appears to have diminished somewhat during the past twelve months, owing to the action of the Charity Commissioners with reference to parish funds, which has deprived the institution of annual subscriptions hitherto paid by the majority of City parishes. That lack of interest in the work of the society is not the cause of this falling off is clear from the fact that the life subscriptions and donations to the society were much in excess of those of the previous year.

Few people probably supposed that the integrity of the philanthropic associations in Russia was great, but few were prepared for the astounding revelations made in a recent number of the *Daily Graphic*. According to their correspondent, the Red Cross Society, which was at first made the general medium of relief distribution, and to which the great bulk of public subscriptions was entrusted, has been robbed by its own administrators of thirteen million roubles. Such a disclosure may well and reasonably make people of this country pause before they give to the Russian relief fund. There are plenty of cases of starvation nearer home, which it is certain money will reach if forthcoming, whereas money given ostensibly to relieve starving Russian peasants may in all probability never reach those for whom it is intended.

The Bishop of London issues an appeal on behalf of the poor of London, who, as he states, have been suffering more from the effects of the prevailing epidemic than they do usually in other winters from the result of snow or frost. He is anxious to mitigate the distress caused by the sickness among the truly poor, whose sufferings are in many cases fearfully aggravated by insufficiency of food, of bed-clothes, and of much-needed nursing. Trouble from sickness, however severe, is mitigated to the wealthy by the comforts and care which their wealth puts at their command; and they cannot better show their sympathy with their less fortunate brethren, who are too often threatened with starvation owing to the illness of the breadwinner of the family, by contributing to the funds of the Metropolitan Visiting and Relief Society, the office of which is at 46A, Pall Mall, S.W.

Now is the season of the year for those who have accumulated stores of old clothes with the intention of giving them away to deserving persons or institutions to remember and carry out their good intentions. Miss Younghusband, of the **GENTLEWOMEN'S EMPLOYMENT CLUB OF WORK AND LEISURE**, will be glad to receive, not only clothing, but books, periodicals, flowers, fruit, vegetables, &c., and donations, however small, to the assistance fund which should be sent to her at 70, Lower Belgrave Street, S.W. In a recent number of the magazine *Work and Leisure*, it was suggested that girls who have leisure might with advantage take up as a hobby the gathering up of the fragments of household stuffs and of dresses, and the converting of them into serviceable articles. In some London parishes this idea has already been carried out with much success by ladies who have formed themselves into a society for the purpose of making up old clothes into useful garments for the poor. Their mode of operation is to meet on certain days, when things which would be thrown away by most persons as useless are made by them into useful articles of wearing apparel.

### SCRAPS AND GLEANINGS.

THE Irish Distressed Ladies' Fund has received a donation of £100 from Mr. R. Benyon.

INFLUENZA seems at last to have touched its highest point of mortality and is now on the wane.

THE total sum for the past year collected by the Bournemouth Hospital Sunday Fund was £611 12s. 3d.

DURING the past year a new wing, "The Alexandra," has been added to the Princess Alice Memorial Hospital, Eastbourne.

AT the annual meeting of the Jenny Lind Infirmary for Sick Children at Norwich, it was proposed to build a new hospital at a cost of £8,000.

A WASHERWOMAN has died in Vienna from influenza, aged 117. Up to her 100th birthday she was regularly at work, and she retained her full vigour to the last.

THE Lord Chancellor will preside at the annual dinner in aid of the Hospital for Sick Children, to be held at the Hotel Metropole, on Friday, March 25th.

A NEW star has suddenly appeared in the constellation Auriga, in the Milky Way. At the Royal Observatory, Greenwich, it was searched for, and found in the position indicated.

THE 66th annual report of the Royal United Hospital, Bath, states that 1891 will stand pre-eminent for the number and gravity of the cases treated. In-patients 1,048 and out-patients 8,448.

AT a meeting of the Council of the Metropolitan Hospital Sunday Fund two anonymous donations of £500 and £50 were announced. It was decided to adopt the uniform system of accounts.

THE appeal for funds to erect a new consumptive hospital at Belfast has been generously responded to. The building and endowment fund now stands £5,550 17s. 8d., as against £695 in January, 1891.

TWO brothers named Hayden, of Carlow, were last May bitten by a rabid dog and immediately sent to Paris, and were treated by M. Pasteur. Last week one brother, aged nine, was taken suddenly ill and died.

DURING the past year 848 children were admitted into the Convalescent Home for Poor Children at St. Leonards. At the annual meeting it was proposed to commence building the sanatorium at once.

THE first of three dances, organised by Mrs. Herring, of Hamilton Place, and Dr. Sibley, of Harley Street, in aid of the North-West London Hospital, will take place at the Portman Rooms on Monday, the 15th inst.

BY the death of Mr. J. R. Hale, the Croydon Hospital, Earlswood Asylum, Reedham Asylum, Aged Pilgrims' Friend's Society, Cancer Hospital, and the Homœopathic Hospital, receive legacies of £1,000 each.

EDISON'S phonograph has been tried by Superintendent Johnson, of the Deaf and Dumb Institute, Indianopolis, U.S. Of 56 boys and girls, 26 could distinguish words, 40 could hear music, and only three of the number heard nothing.

THE Duchess of Bedford has signalised her first public visit to the town of Bedford by distributing the medals and certificates of the St. John Ambulance Association to a large number of ladies and members of the local fire brigade and police force.

THE *Weekly Despatch* declares the late Mr. Holloway left, "among other benefactions, some hundreds of thousands of pounds to the Lord Mayor and Corporation, in trust, to establish a convalescent hospital." It adds that the "will is said to have been wrongfully destroyed."

A MINER named Latus, who is an inmate of the hospital at Myslowitz, in Silesia, has slept since his admission four and a-half months ago. All efforts to wake him have been fruitless. Nourishment, to the extent of two or three litres of milk, is administered daily by a tube inserted into the throat.

THE Throat and Ear Hospital, Newcastle, has, during the past year, treated 2,160 new cases, 517 old cases, making a total of 2,697, and 857 times medicine and advice were given free. The patients, as a testimony to the good work being done, contributed £343 1s. 1d., and the general public subscribed £98.

THE number of patients treated in the Royal Victoria Hospital, Bournemouth, during the past year was 2,784. The receipts were £2,071 10s. 4d., and the expenditure £2,143 19s. 6d. The last instalment of the purchase money (£2,080) has been paid in, and £1,000 borrowed from the furnishing fund paid off.

AT a recent meeting, presided over by the Mayor of Southport, it was proposed to celebrate the centenary of that town by building and endowing a new infirmary. A suitable site has been obtained, and the Mayor (Mr. John Geddes) and Mr. James Wood have generously promised a donation of £1,000 each.

THE Princess of Wales has granted permission for the cot in the Victoria Hospital for Children, which has been endowed by the members of the Children's Salon connected with the *Gentlewoman*, to be named in memory of the Duke of Clarence. Her Royal Highness has selected the words "Albert Victor Edward" to be placed over the cot.

BY command of their Royal Highnesses the Prince and Princess of Wales, Canon Fleming's sermon at Sandringham on the Sunday after the Duke of Clarence's death, entitled "Recognition in Eternity," has been published, price 2s. The profits are to be divided between the Gordon Boys' Home and the British Home for Incurables on Clapham Rise.

IT is reported that some of the natives of a Sussex village are afflicted with the following unusual complaints: One old inhabitant says that her cistern needs supporting—with beef tea and jelly. Another suffers from "nervous nobility;" a third has a brother with indigestion of the lungs and slow fever, and a fourth tells us that his leg is "all somehow"—an admirably descriptive paradox.

THE report for the year ending December 31st last of the Royal Hospital for Children, Glasgow, shows that 481 patients were admitted to the hospital during the year. The average duration of residence was 40·7 and the percentage of deaths was 10·6. The ordinary expenditure of the hospital was £3,291 19s. 2d. Donations for the year amounted to £750. The directors have only £19 13s. 5½d. with which to commence the work of the year.

### The Student of Medicine.

[All communications for this Supplement should be addressed to The Editor of The Hospital, at 140, Strand, with the words, "Students' Column," written in the left hand top corner of the envelope.]

### APPOINTMENTS.

E. Bullar Allan, M.R.C.S., L.R.C.P., appointed House Physician to the Hospital for Consumption and Diseases of the Chest, vice Dr. Stanley. H. Stanley Ballance, M.R.C.S., L.R.C.P., appointed House Physician to the Hospital for Consumption and Diseases of the Chest, vice Dr. Bristowe. H. G. Barling, M B.Lond., F.R.C.S., appointed Consulting Medical Officer to the Tewkesbury Rural Hospital. Henry Draper Bishop, L.R.C.P.Lond., M.R.C.S.Eng., appointed House Physician to the London Hospital, Whitechapel, E. Ernest Dykes Bower, M.R.C.S., L.S.A., appointed Surgeon to the General Infirmary at Gloucester and the Gloucester Eye Institution, vice A. M. Sydney Turner, resigned. Dr. J. E. Fishbourne appointed Fourth Physician to the British Hospital for Mental Disorders. N. Hay Forbes, L.R.C.P.Lond., M.R.C.S., appointed Honorary Surgeon to the Tunbridge Wells Salvage Corps. Harry A. Forsyth, L.D.S.Eng., re-appointed House Surgeon to the London Dental Hospital. J. Harley Gough, L.R.C.P.Lond., M.R.C.S., appointed Honorary Surgeon to the Tavistock Cottage Hospital. H. E. Harris, M.B., L.R.C.P.Lond., M.R.C.S., appointed Medical Superintendent to the St. Saviour's Infirmary, East Dulwich Grove. G. H. Hume, F.R.C.S.Edin., appointed Consulting Surgeon to the Children's Hospital, Newcastle-on-Tyne. F. W. Kennedy, M.D., B.Ch., B.A.O.Dub.Univ., appointed House Surgeon to the Sir Patrick Dun's Hospital, Dublin. F. Lace, L.R.C.P.Lond., M.R.C.S., appointed Honorary Medical Officer to the Eastern Dispensary, Bath. Frederick Page, M.D., appointed Consulting Surgeon to the Children's Hospital, Newcastle-on-Tyne. Eric H. Sharman, M.R.C.S., L.R.C.P., appointed House Surgeon to the Hospital for Sick Children, Great Ormond Street, W.C. George T. Taylor, L.D.S., appointed Dental Surgeon to the Bristol General Hospital, vice T. Cooke Parson, M.R.C.S., L.D.S., deceased.

### VACANCIES.

#### Resident Medical Officers.

Borough Lunatic Asylum, Portsmouth, Clinical Assistant for six months—Board, &c.

District Infirmary, Ashton-under-Lyne, Junior House Surgeon and Dispenser—Salary £50 per annum, board, &c.

East London Hospital for Children, Shadwell, E., Medical Officer, unmarried, appointment for two years—Salary £80 per annum, with board, &c.

East London Hospital for Children, Shadwell, E., House Physician—Board, &c.

General Hospital, Birmingham, House Governor—Salary £300 per annum, with board, &c.

Liverpool Infirmary for Children. Assistant House Surgeon, appointment for six months—Board, &c.

Nottingham General Hospital, Medical Assistant, appointment for six months—Board, &c.

#### Non-Resident Medical Officers.

City of London Hospital for Diseases of the Chest, Victoria Park, E., Assistant Physician; must be Fellow or Member of the Royal College of Physicians, London.

County Kilkenny Infirmary, Second Surgeon—Salary £75 per annum.

Dental Hospital of London, Leicester Square, two Assistant Dental Surgeons; must be Licentiates in Dental Surgery.

London Hospital, Whitechapel, E., Assistant Surgeon.

Queen's Hospital, Birmingham, Casualty Surgeon—Honorarium £50 per annum.

### NOTES FROM THE UNIVERSITIES.

#### Cambridge University.

Lectures on Midwifery.—Dr. W. S. A. Griffith proposes to give an additional course of lectures in midwifery during the present term.

Degrees.—At the Congregation on January 14th the following degrees were conferred: M.B. and B.C.—W. H. Fisher, B.A., Caius; G. Pinder, Queens', R. R. Law, B.A., Christ's.

#### Edinburgh University.

The University was closed on Wednesday, January 20th, by order of the Senators, as a mark of the University's sympathy with the Royal Family on the death of the Duke of Clarence. The Union Ball and the Associated Societies' meeting, when the Marquis of Bute was to deliver his Presidential address, and all other University meetings have been postponed.

At the memorial service on the 20th, held in St. Giles's Cathedral, were present the Senators, Academicians, and the Students' Representative Council of the University, the Royal Colleges of Physicians and Surgeons of Edinburgh, and the Royal Medical Society.

### THE STUDENT OF MEDICINE—(*continued*).

### EDINBURGH PASS LISTS.

#### Royal College of Surgeons.

The following gentlemen having previously passed the necessary examinations and having now attained the legal age, were at the quarterly meeting of the Council on Thursday, January 14th, admitted Fellows of the College: E. Wilkinson, L.R.C.P.Lond., 15, Queen's Terrace, W., diploma of member dated August 2nd, 1888; E. W. G. Masterman, L.R.C.P.Lond., St. Bartholomew's Hospital, diploma of member dated Fdbruary 12th, 1891.

#### Examining Board in England by the Royal Colleges of Physicians and Surgeons.

The following gentlemen passed the Second Examination of the Board in Anatomy and Physiology at a meeting of the Examiners on Monday, January 11th: M. J. Halton, student of Yorkshire College, Leeds; W. W. Hoare, Extramural Academy, Edinburgh; R. J. Roche, Royal College of Surgeons, Ireland; W. Tregenza, of Sheffield Medical School; W. H. Rowlands and J. W. Pridmore, of Queen's College, Birmingham; J. F. Baxter, of Westminster Hospital; and C. F. Druitt, of Bristol School of Medicine and Mr. Cooke's School of Anatomy and Physiology.

Passed in ANATOMY only.—G. G. Oakley, of St. Bartholomew's Hospital; B. Pires, of St. Mary's Hospital; T. H. Agnew, of University College, Liverpool; D. Fogarty, of Ledwich School, Dublin, and Mr. Cooke's School of Anatomy and Physiology; F. A. L. E. Burges, of Queen's College, Birmingham; G. G. Joynson, T. L. Webster, and W. J. Bowden, of Owens College, Manchester; F. Husband, of Bristol Medical School and Mr. Cooke's School of Anatomy and Physiology; W. Mettam, of Sheffield Medical School; M. J. Reubens, of Grant Medical College, Bombay, and Mr. Cooke's School of Anatomy and Physiology; C. Challis, of St. George's Hospital; A. Emlyn, of University College; and A. P. Dantes, of Charing Cross Hospital.

Passed in PHYSIOLOGY only.—W. C. Gent, of Bristol Medical School; E. J. E Coop, H. C. Wood, and K. F. Cherry, of Queen's College, Birmingham; M. Bailey, J. D. McD. Newlands, of University College, Liverpool; E. Brabazon, of Trinity College, Dublin; W. Mansergh, of Owens College, Manchester; R. A. Fegan, of St. Bartholomew's Hospital and Mr. Cooke's School of Anatomy and Physiology; R. H. Power, of St. Thomas's Hospital; J. W. Sweetlove, of Guy's Hospital; and W. B. Murray, of King's College.

Passed in ANATOMY and PHYSIOLOGY on January 12th.—A. F. A. Flower, G. H. W. Ellacombe, and F. W. Gale, of St. Bartholomew's Hospital; S. S. Argyle, of Melbourne University; H. F. W. Armstrong and F. S. Collard, of St. George's Hospital; A. Hair, of University College; F. J. Brakenridge and T. G. Nicholson, of St. Thomas's Hospital; H. R. Walker, of King's College; and A. C. Fry, of Guy's Hospital.

Passed in ANATOMY only.—A. N. Wilde and P. W. G. Shelley, of St. Bartholomew's Hospital: A. S. Matthews, of St. Thomas's Hospital; C. M. Barton, of St. George's Hospital and Mr. Cooke's School of Anatomy and Physiology; and S. A. Francisco, of King's College.

Passed in PHYSIOLOGY only.—M. D. Blake, of Owens College, Manchester, St. George's Hospital, and Mr. Cooke's School of Anatomy and Physiology; E. A. McAnally, of St. George's Hospital; T. S. Farncomb, of Trinity College, Toronto, and Mr. Cooke's School of Anatomy and Physiology; and S. M. Meyrick, of St. Mary's Hospital.

The following gentlemen passed the Second Examination of the Board in Anatomy and Physiology at a meeting of the Examiners on January 13th: C. H. Dunstan, G. S. J. Boyd, and L. L. Burton, students of London Hospital; J. J. Coleman and H. E. Cock, of Guy's Hospital; A. Stanley and J. Ashton, of St. Mary's Hospital; G. S. Pownhall, of St. Bartholomew's Hospital; D. H. Fraser, of St. Thomas's Hospital; J. C. Clemesha and R. A. Bowie, of McGill University, Montreal.

Passed in ANATOMY only.—N. B. Baker, of St. Bartholomew's Hospital; A. Carruthers and W. J. C. Ray, of St. Thomas's Hospital; P. J. Curtis, of Guy's Hospital; A. K. M. Curtis and R. Sim, of Middlesex Hospital; A. A. Lewin, of McGill University, Montreal; and A. R. S. Freeland, of London Hospital.

Passed in PHYSIOLOGY only.—W. G. Noble, of London Hospital; B. P. O'Neill, of Guy's Hospital; F. A. W. Quay, of Trinity College, Toronto; and H. C. Clark, of Middlesex Hospital.

Passed on January 14 in ANATOMY and PHYSIOLOGY: W. H. Bracewell, of Melbourne University; R. Lawson, of St. Thomas's Hospital; E. F. G. Tucker, J. D. Galloway, and H. W. Gratton, of London Hospital; E. W. de Kretser, of Ceylon Medical College; R. C. Griffiths, of Toronto University; L. W. Oliver, of St Mary's Hospital.

Passed in ANATOMY only.—W. C. F. Hayward and F. A. L. Hammond, of Charing Cross Hospital; W. Mawer, H. W. Carson, and M. R. Taylor, of St. Bartholomew's Hospital; C. H. Wilmer, of St. Bartholomew's Hospital and Mr. Cooke's School of Anatomy and Physiology; D. F. Mannsell, of St. Thomas's Hospital; F. E Bromley, W. V. P. Teague and S. H. L. Archer, of London Hospital; F. Romer, of St. George's Hospital and Mr. Cooke's School of Anatomy and Physiology; G. S. S. Marshall, of Middlesex Hospital; E. G. Frederick, of King's College; and W. H. Chute, of Westminster Hospital.

Passed in PHYSIOLOGY only.—P. L. Moore, of Cambridge University; G. M. Bennett, L. A. Williams, and E. D. J. O'Malley, of Middlesex Hospital; A. W. Lamb and J. E. Jones, of St. Bartholomew's Hospital; R. Slocock, V. Graham, A. D. Cowburn, and S B. Stedman, of St. Thomas's Hospital; J. A. W. Pereira, of Bombay and Charing Cross Hospital; W. L. Roberts and J. N. Hall, of St. Mary's Hospital; D. Evans, of University College; J. Heard, of Westminster Hospital; L. Benstead and F. H. L. Cloud, of Guy's Hospital.

One hundred and six candidates presented themselves in Anatomy and Physiology, of whom thirty-two were referred in both subjects for three months, and four for six months. Twelve were referred in Anatomy only, and nineteen in Physiology only.

### THE STUDENT OF MEDICINE—(*continued*).

Twenty-seven candidates presented themselves in Anatomy only, of whom five were referred.

Thirty-seven candidates presented themselves in Physiology only, twelve of whom were referred.

**Royal Colleges of Physicians and Surgeons of Edinburgh, and Faculty of Physicians and Surgeons of Glasgow.**

The quarterly examinations in Edinburgh for the Triple Qualification took place in January and February, with the following results :—

First Examination :—

Of 52 candidates, the following 34 passed :—T. C. Smith, Victoria (with honors), W. Morris, J. Ingram, J. G. Portal, C. W. Lawson, H. Highet, T. G. G. Symons (with honors), G. W. Baker, J. Meade, T. Murphy, D. G. Newland, J. J. Harvey, B. Shreenivassa, A. P. Dias, W. Gordon, M. Breen, G. H. Monkhouse, A. J. Young, J. W. Lax, E. B. H. Hughes, J. Wishart, J. T. Jones, J. K. Brownlees, F. Smith, A. Pearson, W. Pole, J. Minns, J. O'Sullivan, J. Waddell, J. A. Humphrey, G. H. K. Ross, E. F. O'Ryan, S. Robb, and H. S. O'Conor.

The following candidates passed in the respective divisions :—

Chemistry.—J. A. Sanderson, D. J. M. Vallange, and J. B. Voortman.

Elementary Anatomy and Histology.—R. P. Graham.

Elementary Anatomy.—S. Potter.

Second Examination.

Of 59 candidates, the following 30 passed : Minnie E. Bowlby, E. T. Whitaker, R. L. Park, G. Moore, C. S. Dudgeon, L. W. Harvey, M. Donovan, D. G. Newland, A. G. Rolland, E. R. A. MacDonnell, G. W. Beesley, J. G. Thomson, M. P. Corkery, W. Bell, J. F. O'Meara, G. H. K. Ross, D. R. Taylor, S. I. Williams, H. Jan Van Brockhinzen, T. J. Kennedy, S. Gawn, T. Keays, J. H. Wilson, A. T. Brown, R. H. Manson, L. T. Myers, T. M. Donovan, J. C. Loughridge, F. C. Rundle, and J. Morrison.

The following candidates passed in the respective divisions :—

Anatomy.—W. H. Ferrier.

Physiology.—H. W. Fisher.

Materia Medica and Pharmacy.—S. H. Noble and J. S. D. Mac Cormac.

Final Examination.

Of 121 candidates, the following 57 passed, and were admitted L.R.C.P. and S E., and L.F.P. and S.G. : J. A. Ross, G. H. S. Hillyar, L. Whitby, S. Firth, P. W. McGregor, F. W. Toms, R. E. Roberts, A. A. Wilson, E. M. Harris, F. P. Bassett, A. K. Jarratt, S. W. Thompstone, T. L. Johnston, R. M. Cooper, R. Martin, E. H. Hackett, R. D. Stokes, B. Harris, W. K. Inksetter, H. D. Hamilton, J. Ross, J. L. Smith, P. Q. Ambrose, R. T. Davies, W. C. Rainsbury, Martha G. I. Cadell, G. M. Stocks, A. J. Millar, C. J. Johnston, S. McNair, A. L. L. Chignell, H. A. Smit, R. Holmes, T. M. Bassano, A. W. Robertson, H. A. Cruthwell, J. Cumming, P. A. Conran, H. Webster, W. H. Stephenson, E. F. W. Moon, G. B. Mitchell, W. Leahy, W. J. Baird, R. Muschamp, R. H. Griffith, B. Keane, G. Greswell, A. G. Jackson, H. F. J. Graves, W. G. Sellar, J. J. Moynihan, C. J. Caregan, R. MacCarthy, G. H. Jenkins, G. R. Fortune, and H. A. L. Howell.

The following candidates passed in the respective divisions :—

Medicine and Therapeutics.—F. A. Godfrey, J. Wilkie, and W. S. Howard.

Surgery and Surgical Anatomy.—C. A. Brough (with honours) and R. McConll.

Midwifery and Medical Jurisprudence.—P. I. de Villiers.

Surgery and Surgical Anatomy, Midwifery, and Medical Jurisprudence.—G. Hepworth.

### ATHLETICS.

### Football.—Rugby : Hospital Cup Ties.

The first round results are :—

Guy's beat Charing Cross, five goals five trials to nil, January 26th.

University beat St. Mary's, two goals three tries to one try, January 28th.

London v. St. Thomas's (postponed).

St. Bartholomew, Middlesex, Westminster, King's, and St. George's byes.

Draw for the Second Round.—Guy's v. St. Thomas's or London, University v. Middlesex, King's v. St. George's, St. Bartholomew's v. Westminster. The dates will be duly announced.

Saturday, January 30th.

St. George's Hospital beat Pritchard's by two goals and two tries to one goal and one try.

St. Mary's Hospital beat Wasps by a goal to two tries.

St. Thomas's Hospital beat Civil Service by six goals and eight tries to nil.

Rosslyn Park v. Guy's Hospital.—Played at Acton, the Rosslyn Park team winning by two goals and two tries to nothing.

Old Paulines beat London Hospital by three goals and one try to nil.

Middlesex Hospital beat Lennox by one goal to two tries.

Association Challenge Cup, January 28th.

St. Bartholomew's v. St. George's.—Won by former by 10 goals to love.

Casuals v. King's College Hospital.—Draw 2 goals each.

January 30th.

Swindon Town v. London Hospital.—Played at Swindon, and won by the locals by 9 goals to 2.

Middlesx Hospital beat Acton by 3 goals to nil.

Old St. Stephen's v. St. Mary's Hospital.—Played at Merton Hill, Wimbledon, and resulted in favour of the Old Boys by 3 goals to nil.

Reigate beat St. Bartholomew's Hospital by 5 goals to 1.

Inter Hospital Association Cup.—(Second Round.)

St. Bartholomew's Hospital beat University Hospital by six goals to one.

